# Recruiting faith- and non-faith-based schools, adolescents and parents to a cluster randomised sexual-health trial: experiences, challenges and lessons from the mixed-methods Jack Feasibility Trial

**DOI:** 10.1186/s13063-016-1506-y

**Published:** 2016-07-29

**Authors:** Áine Aventin, Maria Lohan, Lisa Maguire, Mike Clarke

**Affiliations:** 1 School of Nursing & Midwifery, Queen’s University Belfast, 97 Lisburn Road, Belfast, Northern Ireland; 2 Centre for Public Health, School of Medicine, Dentistry and Biomedical Sciences, Queen’s University Belfast, Belfast, Northern Ireland

**Keywords:** Recruitment, Schools, Randomised controlled trial, Cluster randomised controlled trial, Sexual health, Complex interventions, Barriers and facilitators

## Abstract

**Background:**

The move toward evidence-based education has led to increasing numbers of randomised trials in schools. However, the literature on recruitment to non-clinical trials is relatively underdeveloped, when compared to that of clinical trials. Recruitment to school-based randomised trials is, however, challenging, even more so when the focus of the study is a sensitive issue such as sexual health. This article reflects on the challenges of recruiting post-primary schools, adolescent pupils and parents to a cluster randomised feasibility trial of a sexual-health intervention, and the strategies employed to address them.

**Methods:**

The Jack Trial was funded by the UK National Institute for Health Research. It comprised a feasibility study of an interactive film-based sexual-health intervention entitled *If I Were Jack*, recruiting over 800 adolescents from eight socio-demographically diverse post-primary schools in Northern Ireland. It aimed to determine the facilitators and barriers to recruitment and retention to a school-based sexual-health trial and identify optimal multi-level strategies for an effectiveness study. As part of an embedded process evaluation, we conducted semi-structured interviews and focus groups with principals, vice-principals, teachers, pupils and parents recruited to the study as well as classroom observations and a parents’ survey.

**Results:**

With reference to social learning theory, we identified a number of individual-, behavioural- and environmental-level factors that influenced recruitment. Commonly identified facilitators included perceptions of the relevance and potential benefit of the intervention to adolescents, the credibility of the organisation and individuals running the study, support offered by trial staff, and financial incentives. Key barriers were prior commitment to other research, lack of time and resources, and perceptions that the intervention was incompatible with pupil or parent needs or the school ethos.

**Conclusions:**

Reflecting on the methodological challenges of recruiting to a school-based sexual-health feasibility trial, this study highlights pertinent general and trial-specific facilitators and barriers to recruitment, which will prove useful for future trials with schools, adolescent pupils and parents.

**Trial registration:**

ISRCTN 11632300. Registered on 19 December 2014.

## Background

Recruiting adequate numbers of participants to randomised controlled trials (RCTs) and retaining them for the entire duration of a study, while challenging, is essential for internal and external validity and minimising bias, which can be introduced when certain groups of individuals refuse participation. While the process of randomisation eliminates selection bias [1], cluster randomised trials, in which groups are randomised rather than individuals, may be more susceptible to bias, with one systematic review finding 40 % with identifiable biases [2]. This highlights that extreme care needs to be taken in the design and recruitment of such trials.

While slight improvements have been reported in recent times [3], recruitment to RCTs remains a problematic issue [4]. As many as 45 % of publicly funded RCTs do not reach their recruitment targets, with almost half requiring an extension due to recruitment difficulties [3]. The development of methods to improve recruitment is, therefore, a top priority for trial methodologists [5].

In a recent systematic review of methods to improve recruitment to clinical RCTs, Treweek, et al. [6] concluded that effective strategies include: (i) the use of opt-out rather than opt-in procedures, (ii) telephone reminders to non-respondents and (iii) open designs, which permit participants to know which treatment they are receiving in the trial. While the impact varies across studies, others have reported recruitment facilitators to include involving the target population in developing the intervention and preparing participant information about the study [7]; a personalised and culturally sensitive approach to potential participants including methodological innovations that pay attention to participant contact and convenience, incentives and human factors such as relationships [4, 8–10]; recruitment processes that highlight the beneficial outcomes of taking part and address any barriers or perceived negative outcomes [11]; providing research and implementation support through a dedicated research team contact [10]; and providing monetary incentives [4, 12]. While some have suggested the value of using online and mobile technology for improving recruitment [13–16], Treweek, et al. [6] concluded that their effect is not yet clear. More research in this area is likely warranted as there may be advantages in providing information in this way, such as, increasing credibility and engagement with trial information.

Even though some studies have reported barriers and facilitators of recruitment specific to school-based RCTs [9, 11, 12, 17–19], there is insufficient knowledge regarding the factors influencing recruitment to *non-clinical* trials, such as those conducted in schools, when compared to published information on the successful conduct of *clinical* trials. It is generally agreed, however, that major impediments to the recruitment of schools include excessive demands on schools to take part in research and participant perceptions of the potential extra burden of research within the already busy school context and overcrowded academic curriculum [9, 11, 17]. In an attempt to address such problems, researchers in Wales and England have developed School Health Research Networks (www.uclpartners.com/our-work/academic-health-science-network/integrated-children-young-people-and-maternal-health/schools-research-network, http://www.shrn.org.uk/), which aim to improve the quality and relevance of health research in schools and create a sustainable network of schools that are research-ready yet not overburdened. While there are several possible benefits of such networks, evaluations of their long-term feasibility and sustainability have yet to be reported. Furthermore, potential limitations may emerge from restricting the pool of schools and/or researchers committed to involvement in research within a particular country.

Other challenges to recruitment in school-based trials emerge when the focus of the research is a sensitive topic, such as sexual health. In such instances, gatekeepers, such as school management and parents, may be understandably concerned about any potential negative impact on pupils and, in some schools, whether the research fits with their particular religious ethos. Some UK-based sexual-health studies have responded to such potential obstacles by excluding denominational schools (in particular Catholic schools) [20, 21]. While this risks decreasing the representativeness of the sample, it may be necessary given the challenges involved in engaging such schools in sexual-health trials.

Feasibility trials, referred to as phase II trials in the UK Medical Research Council Framework for complex interventions [22], which are intended as precursors to effectiveness (phase III) RCTs, offer opportunities to examine challenges to successful recruitment and explore possible solutions in the particular context in which a trial is taking place. The Jack Feasibility Trial was a 2-year project funded by the UK National Institute for Health Research (NIHR), which began in May 2014. It was a cluster randomised feasibility trial with embedded process and economic evaluations, recruiting over 800 adolescents, relationship and sexuality education (RSE) teachers, other school staff and parents in eight socio-demographically diverse post-primary schools in Northern Ireland (NI). Four schools, randomly assigned to the intervention group received the 4-week *If I Were Jack* intervention [23] and four schools randomly assigned to the control group continued with normal RSE practice. All pupils were asked to complete a questionnaire at baseline and 5 and 9 months later [24], and parents and guardians of pupils in the intervention group were invited to attend a 1-hour information and discussion session facilitated by the schools. As well as estimating recruitment and retention rates for a future effectiveness trial, the study aimed to determine the barriers and facilitators of recruitment to a school-based sexual-health trial and identify optimal strategies for recruiting schools (including Catholic schools), pupils and, in line with research that suggests the important role they play in adolescent sexual-health outcomes [25, 26], parents and guardians. This article describes the challenges of recruiting to the trial and the strategies we adopted in an attempt to address them.

## Methods

### Recruitment targets and protocols

The aim was to purposively recruit eight post-primary schools, stratified by management type and deprivation, with at least one teacher willing to facilitate implementation of the intervention and/or data collection. We sought to involve all year 11 pupils aged between 14 and 16 attending the school and their parents or guardians, and a sample of school staff, to take part in semi-structured interviews regarding the acceptability and feasibility of participation in the trial. Informed consent was obtained from all participants. The trial was overseen by an independent trial steering committee and was registered prospectively (ISRCTN 11632300). All initial approaches to school principals and oral presentations of trial information to teachers and pupils were conducted by the first author in her role as trial manager.

#### Schools

In 2013, there were 201 eligible post-primary schools in NI, which can be broadly categorised as secondary (*n* = 133) or grammar (*n* = 68). The primary difference between the two categories is that while all pupils can attend secondary schools, only those who demonstrate educational attainment by passing an entry exam can obtain places in grammar schools. Various management structures also exist, with controlled schools (*n* = 75) managed by the Education Authority of NI and voluntary and maintained schools (*n* = 135) managed by a board of trustees. The Catholic Church manages a significant number of voluntary Catholic maintained secondary (*n* = 68) and grammar (*n* = 29) schools. In NI, although religion is not a criterion for attendance, most pupils at controlled schools are from Protestant denominations and most attending Catholic maintained schools are Catholic. There are also 20 integrated schools, which aim to provide a religiously and culturally mixed environment.

Reflecting our aim to capture the acceptability and feasibility of the intervention and research process in this diverse educational context, we initially stratified our sample according to school management type and deprivation (indicated for the purposes of the study by the percentage of pupils at the school eligible for free school meals). We anticipated potential challenges in recruiting Catholic schools since some may perceive an abstinence-plus intervention such as *If I Were Jack* as incompatible with the Catholic ethos (the intervention is abstinence-plus in the sense that it refers to issues such as contraception and abortion as well as abstinence; see [23]). Although a number of recent UK-based school-based sexual-health trials did not attempt to recruit faith-based schools [20, 21, 27], we thought it was important to try to include them, given the large numbers of Catholic schools in NI and the lack of published information on the feasibility of recruiting such schools to sexual-health trials.

Conversely, we anticipated that there would be greater uptake of the intervention in deprived areas where teenage birth rates are generally much higher [28]. We, therefore, aimed to recruit two Catholic grammar schools and two schools in deprived areas and randomly assign one of each to the control and intervention groups. We had no difficulties recruiting schools in deprived areas and, contrary to expectations, few difficulties recruiting Catholic maintained secondary schools (which do not academically select pupils), but we had significant problems recruiting Catholic grammar schools and voluntary other-managed grammar schools (such schools are usually privately funded and managed by a board of governors). Due to these difficulties and looming deadlines, we revised our stratification definitions to recruit the following: two secondary schools of any management type in deprived areas, two controlled secondary schools, two Catholic schools and two grammar schools. Schools were the unit of randomisation and, after baseline data collection, the schools were grouped into four pairs (secondary schools in deprived areas, Catholic schools, grammar schools and other types) and randomised to ensure that one of each pair was assigned to the intervention group.

We used three strategies to recruit targeted schools: (i) RSE teacher training events, (ii) personal introductions by members of advisory and steering groups and (iii) cold-call invitation. We chose recruitment at statutory RSE training days as our primary recruitment strategy because we thought that it would optimise the potential to recruit schools that saw RSE as a priority subject and/or had an interest in developing their RSE curriculum. Additionally, we thought that the opportunity to promote the intervention and research process among key school stakeholders at a government-funded training event and by addressing any concerns in situ, would lead to positive perceptions of the credibility of the research team and the benefits of involvement in the trial. Upon request to the facilitators, we were invited to give a 30-minute recruitment presentation at two of these events. We introduced the intervention and provided an overview of the research. This included the presentation of a 5-minute video of health and education experts talking about the intervention and its potential benefits for use in the classroom, as well as testimonials from teachers who had used it during the pre-piloting development phase. Teachers in attendance were asked to provide their contact details if they were interested in receiving more information about the research. The schools that we approached following these events (via letter of invitation to the principal and follow-up phone call) were selected on the basis of expressed interest and fit with our recruitment criteria. Schools attending the event meeting these criteria that declined to participate after the initial contact were replaced by the next school from the list of all eligible schools attending the event.

We also used a second strategy – recruitment following introduction by the trial’s steering and advisory group members – as an aid to recruiting our target of two Catholic grammar schools and one voluntary other-managed grammar school, both of which we had difficulties recruiting and had low attendance of representatives at the RSE training events. We asked steering and advisory group members who had contacts in post-primary schools if they would be willing to suggest schools that might be amenable to receiving information about the research or to contact a representative of a potential school introducing the trial manager and seeking an expression of interest in receiving a letter of invitation and follow-up phone call from the research team.

Finally, we attempted recruitment through cold-call invitation. In addition to including the standard information sheet (which contained minimal information on the intervention), we included a flyer with promotional detail on the Jack intervention emphasising that this was a fully prepared off-the-shelf resource, testimonials from experts and end users, and a link to the project website as well as a letter of invitation, which stated that we had ‘one place remaining’ for a Catholic grammar or voluntary other managed school. All formal letters of invitation were followed up within a week of sending with a phone call to the school principal from the trial manager.

#### Pupils

The target population was all year 11 pupils aged 14 to 16 in participating schools. Pupils were excluded if their parents/guardians withdrew them from the study by returning opt-out forms to the research team; if they themselves declined to take part; or if they were unable to understand the research documentation because English was not their first language. Pupils with reading difficulties and/or learning disabilities were offered the opportunity to have the research documents read aloud by their teaching assistant or a research assistant. Based on the average size of year 11 class groups in schools in 2011/12 (mean 114; median 113) and allowing for an 80 % consent rate, we estimated that we would recruit approximately 730 pupils to the study.

Teachers were provided with information sheets to distribute, 1 week prior to baseline data collection, to year 11 pupils whose parents had not withdrawn them from the study. They were asked to encourage pupils to read the information sheet and inform them that they would have an opportunity to ask questions about it and decide whether or not they wanted to take part before completing the questionnaire the following week. At the beginning of the baseline data collection sessions, either the trial manager or a trained research assistant spent 15 minutes explaining the research to pupils, giving them an opportunity to ask questions and asking them to complete a consent form indicating whether or not they wanted to take part. Although pupil questions were not formally recorded, pupils generally sought clarification of the concepts of anonymity and confidentiality, i.e. reassurance that parents and teachers would not be able to read their responses. Some pupils also asked about what would happen to the results and what the benefits of participation were for them.

#### Parents and guardians

We sought to recruit at least one parent or guardian of each participating year 11 pupil in the intervention group to attend a 1-hour parents’ and guardians’ information and discussion session at their child’s school. Based on reports from teachers regarding difficulties engaging parents in non-academic activities, we estimated that parent/guardian representatives of approximately 50 % of year 11 pupils would attend these sessions, which we projected would result in around 200 participating parents. From those who attended these sessions, we aimed to recruit approximately 25 to take part in focus group interviews with a researcher. In an attempt to reach those who did not attend the sessions, we recorded a 6-minute video containing key messages and posted it on YouTube, sending a closed link to parents/guardians via text message. Following the implementation, parents and guardians of pupils in intervention schools were sent a text message containing a link to a short survey, which asked them for their views on the intervention. Respondents were entered in a prize draw for £100. Parents and guardians of pupils in the control group were not recruited to the study.

### Process evaluation

#### Data collection

Data collection for the process evaluation consisted of two elements:

i. Recruitment rates: We recorded the number of expressions of interest at RSE training days, invitation letters sent, responses received, telephone calls made, emails sent, participants declining participation, and participants agreeing to take part in the study.
ii. Barriers and facilitators to recruitment: We collected qualitative data from a combination of field notes, records of email communication and contact notes following telephone conversations with participants who declined to participate, documented meetings with steering group members, and semi-structured interviews with principals, vice-principals, teachers and parents recruited to the study. Interviews were conducted by two female postdoctoral research fellows, AA and LM, both of whom have experience in conducting school-based research. Participants were informed that the goal of this element of the research was to record their experiences of the recruitment process (both positive and negative) so that we might plan for a larger trial.

#### Data analysis

i. To calculate recruitment rates, we derived a percentage from the total number of invitations sent by school management type and the resulting number of schools, pupils and parents recruited.
ii. Qualitative data were organised using *NVivo 10* and analysed using a form of thematic analysis based on the six steps proposed by Braun and Clarke [29]. This involved moving between inductively derived codes emerging from the data and searching for data on predefined themes outlined in our topic guides. These inductively and deductively derived codes were independently analysed by two research team members (AA and ML) to form overarching themes.

## Results

### Recruitment rates

**Fig. 1:**
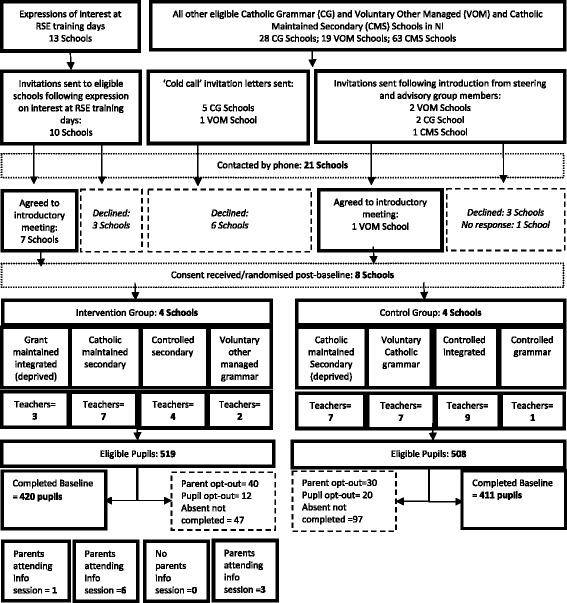
Jack trial CONSORT recruitment flow diagram. *CG* Catholic grammar school, *CMS* Catholic maintained secondary school, *RSE* relationship and sexuality education, *VOM* voluntary other-managed school

A total of eight schools, six principals, two vice-principals, 40 teachers and 831 pupils were recruited at baseline. In intervention schools, ten parents attended the parents’ information sessions, 45 watched the YouTube video, eight took part in a semi-structured interview and 29 responded to the parents’ survey (see Fig. 1).

#### School recruitment rates

Recruitment of schools via RSE training events was by far the most successful strategy, leading to 70 % of schools (*n* = 7) invited being randomised into the study. Invitation following introduction by a steering or advisory group member led to the recruitment of one additional school (a 20 % recruitment rate). The cold-call invitation was unsuccessful, with all six schools approached declining to take part.

As illustrated in Table 1, our main challenge was in recruiting Catholic grammar schools (i.e. Catholic schools that use academic ability to select pupils) and voluntary other-managed grammar schools (i.e. non-Catholic Church or privately funded schools that use academic ability to select pupils). While the overall school recruitment rate was 38 % (100 % for integrated, controlled grammar and controlled secondary schools), the recruitment rates for Catholic maintained secondary schools, Catholic grammar schools and voluntary other-managed grammar schools were 67 %, 13 % and 17 %, respectively.

**Table 1:** School recruitment

	Integrated	Controlled secondary	Catholic maintained secondary	Controlled grammar	Voluntary Catholic grammar	Voluntary other-managed grammar	All
Number of schools contacted	2	1	3	1	8	6	21
Number of schools recruited	2	1	2	1	1	1	8
School recruitment rate (schools recruited / contacted)	100 %	100 %	67 %	100 %	13 %	17 %	38 %

#### Pupil recruitment rates

Recruitment of 831 pupils to the study at baseline represents an overall pupil recruitment rate of 80.9 %. Parental withdrawal of consent accounted for 6.8 % of the loss (*n* = 70) and pupil opt-out for 3.1 % (*n* = 32). Pupil absence or unavailability at baseline with absentee questionnaires and consent forms not returned to the research team accounted for the remaining 9.1 % of loss (*n* = 94) (Table 2).

**Table 2:** Pupil recruitment by school type

	Integrated		Controlled secondary	Catholic maintained secondary		Controlled grammar	Voluntary Catholic grammar	Voluntary other-managed grammar	All
Number of eligible pupils	132	121	91	98	149	123	155	158	1027
Number of parental withdrawals of consent	12	11	2	2	9	10	6	18	70
Number of pupil opt-outs at baseline	17	10	0	2	2	1	0	0	32
Number of pupils absent at baseline^a^	0	24	5	6	2	40	1	16	94
Number of pupils recruited at baseline	103	76	84	88	136	72	148	124	831
Pupil recruitment rate at baseline^b^	78.0 %	62.8 %	92.3 %	89.8 %	91.3 %	59.3 %	95.5 %	79.1 %	80.9 %

^a^Absent at baseline and questionnaire not completed/returned

^b^Recruited pupils / eligible pupils

#### Parent/guardian recruitment rates

Recruitment of parents and guardians to attend the school-facilitated information and discussion session was extremely low, with an overall recruitment rate of 2.3 % (i.e. nine mothers and one father representing ten different pupils) assuming potential recruitment of one representative parent or guardian for each participating pupil in the three intervention schools that held the session (*n* = 428). We suggested that schools run the event in the evening to facilitate attendance by working parents; however, all chose to run the event in the late afternoon (two schools started at 3.30 pm and one at 4.30 pm). Reasons for this early start included standard practice for schools to hold events at these times, difficulties in keeping the school open late for such an event and a lack of desire on the part of the teachers to stay late themselves. One school did not hold the parents’ session because the teacher who was responsible for facilitating it went on sick leave and decided it was no longer feasible to organise upon her return one week later. Altogether, 45 parents viewed the information session video on the YouTube channel (12.5 % response rate) and 29 parents responded to the parent’s online survey (8.3 % response rate).

### Reasons for participation and non-participation

#### Schools

A total of 13 schools approached declined to participate in the study (Table 3). All but one of these schools made this decision before meeting with the trial manager. The most common reason for non-participation (*n* = 4) was that, having considered the information sheets, school management and/or the teacher responsible for delivering *Learning for Life and Work* considered that there was no time available within the current curriculum at year 11 to deliver the intervention and take part in the research. Three schools also reported that they were already involved in another research project and could not accommodate a further study at that time. Two other schools indicated that, due to staff changes (maternity leave and a change in the *Learning for Life and Work* team), they did not think it was an appropriate time to take part. Two schools declined to take part without providing a reason. One Catholic grammar school declined to take part having consulted with the school chaplain, who felt that the intervention was not compatible with the school ethos. Although the administrators/secretaries in four schools mentioned at first contact that they did not have any concerns about teenage pregnancy in their school, only one principal/RSE contact in these schools cited this as their primary reason for non-participation.

**Table 3:** Reasons for non-participation by school type

	Number of Catholic maintained secondary	Number of voluntary Catholic grammar	Number of voluntary other-managed grammar	All school types
No space in LLW curriculum	1	2	1	4
Currently involved in other research	0	2	1	3
Not a suitable time due to staff changes	0	1	1	2
Declined without providing reason	0	2	0	2
Intervention incompatible with ethos	0	1	0	1
Intervention incompatible with needs	0	0	1	1
All non-participating schools	1	8	4	13

*LLW Learning for Life and Work*

Conversely, the schools that did participate in the research reported doing so for a number of reasons (see Table 4), primary among which was their desire to improve RSE provision for their pupils (*n* = 6) and their belief that the *If I Were Jack* resource would be of benefit to their pupils (*n* = 8). Schools in deprived areas were particularly interested in taking part because of their current and past experiences of teenage pregnancy in the community (*n* = 5). One school noted that participation in the research would also look good at an upcoming inspection and another school felt confident that they would benefit because of previous positive experiences of research participation. All principals also mentioned that the credibility of the research had been a deciding factor (*n* =8).

**Table 4:** Reasons for school participation

	Number (%)
Belief that the intervention would be useful to pupils	8 (100 %)
Credibility of the intervention/research	8 (100 %)
Desire to improve RSE provision within the school	6 (75 %)
Compatibility of the intervention with pupil needs (teenage pregnancy a concern)	5 (63 %)
Positive experiences with previous research	1 (13 %)
Involvement in research perceived as beneficial for upcoming inspection	1 (13 %)

Principals indicated that they were approached several times a week to take part in research, and while all appreciated the potential benefits of research, they were often too busy to respond to such requests. All agreed that direct face-to-face contact with them or a subject teacher was the optimal strategy:

*If [an invitation] just comes as an email there is a fair chance it’ll end up in the bin […] If it’s preceded by a phone call there is probably a better chance [it’ll be considered]*. (Principal, Catholic maintained secondary)

*If you just send something in and it comes to me, it could go over my head. If you send it to my teacher or you engage somebody or you phone the school up and say ‘Could I come in to chat to the Principal?’ or ‘Could I come in to speak to somebody?’ rather than sending a letter, I think that’s the way to hook people in*. (Principal, integrated)

Principals agreed that a number of considerations were important in helping them to make a decision about whether or not to take part. Central among these were the potential burden on pupils, especially those in exam classes, the burden on teachers and the credibility of the project:

*Firstly, if there’s any credibility in what’s being done. Secondly, if it can be fitted in with minimum disruption. If it’s going to affect exam classes there’s almost no chance*. (Principal, Catholic maintained secondary)

*R: What kind of things do you consider before you decide whether or not you want to take part?*

*P: Well, the first thing I would look at is, is it in an area of interest that we can contribute to that is very specific to my school? Is it an area that the students can benefit from? So, with the Jack project, I felt it was a wee bit innovative and that there were potential useful resources from it. The [other] things that I look at are what are the time constraints and the commitment for the students and the staff?* (Principal, integrated)

Principals especially did not want pupils in significant exam years to be disrupted with external research studies:

*If you’re wanting to do it with year 11 to 14, especially year 11 and 12, there’s hardly a week goes by where they’re not involved in some kind of controlled assessment […] I won’t take kids out of English and Maths or maybe even other GCSE subjects to do [research]*. (Principal, voluntary other-managed grammar)

Principals also had an especially strong message for researchers in terms of taking some of the responsibility for the organisation of the research within the school, especially in terms of conducting the data collection:

*[I would immediately decline to take part in research projects] that are going to be very time-consuming … or, where projects put the onus on the school – ‘Could you get us a group? Here’s the list – could you go away and do it, and when you’re finished, could you bring it back to us and I’ll pick it up from you?’ and you’re like ‘What?!’* (Principal, integrated)

Principals agreed that monetary incentives would be an important facilitator of participation:

*R: How important are monetary or other incentives, such as getting the Jack resource to use at the end?*

*P: Very important. They would be the enticement to get you involved. It sounds very mercenary and I don’t mean to be mercenary in this day and age, but if I’m going to… if it’s going to be time and energy with students and staff and commitment, and if the benefits are not solely related to students and staff and outcomes, then there’s got to be a reason why you would do it*. (Principal, integrated)

*I think more schools would take [participation] into consideration, especially given how tight budgets are getting*. (Principal, Catholic maintained secondary)

*If somebody was coming in and saying […] ‘If you take part in this, we’re going to give you a thousand pounds’, you know, a thousand pounds directed to one specific thing to support pupils in school is quite a lot of money. But if somebody comes in and says, you know, ‘We’ll give you some money for this – here’s a hundred pounds’, well, that’s not going to be an incentive one way or the other.* (Principal, voluntary other-managed grammar)

### Pupils

Pupil participation varied by school type (see Table 2) with the highest recruitment rates in the three Catholic schools (>90 %) and the controlled secondary school (92.3 %). The lowest rates were in the controlled grammar school (59.3 %) and one of the integrated schools (62.8 %), both of which had high pupil absentee rates on the day of baseline data collection. The controlled grammar school’s absentee numbers (*n* = 40) were because one class group were not permitted to leave their class to take part due to an observation for teacher training purposes and the remaining pupils were attending an unanticipated sports event. In the integrated school, the absences (*n* = 24) were because data collection took place first thing on a Monday morning when absences were usually higher. Trial champions were provided with questionnaires and consent forms for absent pupils and asked to return them on several occasions before a specified cut-off point 1 month later. Teachers reported an inability to find time to administer the questionnaires to pupils as the reason for not returning them.

Pupil opt-out rates ranged from 0 to 13 % across the schools, with the highest rates in both integrated schools (see Table 2). Although space was provided on the questionnaire for comments, pupils were not required to give a reason for non-participation. Observations by the research team delivering the questionnaires indicated that some pupils chose not to participate because they wanted to focus on other school work at the time of data collection, others thought the questionnaire was too long and some did not feel comfortable answering questions relating to sexuality. We also observed that pupil opt-outs tended to occur in friendship groups (i.e. it was rare for a single young person to opt-out, rather pairs or groups of friends tended to opt-out together). In the school with the highest opt-out rate (*n* = 17), teachers had not provided pupils with the information sheet before the study and baseline data collection was scheduled to follow straight after an exam in a large exam hall, which made it very difficult to provide an overview of the study at a high enough volume for all to hear. In the words of one of the attending teachers, decisions to opt-out ‘spread like wildfire’ in one area of the hall.

As indicated in Table 2, parental withdrawal of consent rates ranged from 2 to 11 %, with the lowest rates in one of the Catholic maintained secondary schools and the controlled secondary school (*n* = 2, 2 % in both) and the highest in the voluntary other-managed grammar (*n* = 18, 11 %). Most parents did not include a reason, but of those who did (*n* = 16), the reasons provided were variations of ‘My child does not want to take part’ (*n* = 10), ‘I do not want my child to take part’ (*n* = 2), ‘My child wants to focus on his/her exam subjects’ (*n* = 1), ‘My child is busy with extra-curricular activities’ (*n* = 2) and ‘My child has a learning disability so I do not think it is appropriate for him to take part’ (*n* = 1).

At the time of data collection, teachers in four of the schools reported that some parents had been in contact with them because they were confused about whether or not to send back the withdrawal of consent form if they were happy for their child to take part. In the voluntary Catholic grammar school, five parents who had initially opted out contacted the school after their child had received the information sheet to say that they were now happy for their child to take part. Teachers theorised that this may have been at the request of the child but one teacher reported that a parent she had spoken to had previously thought that her child was being ‘singled out’ for participation in the study but changed her mind when she realised everyone would be taking part.

### Parents and guardians

Altogether 29 parents/guardians responded to an online survey, with 22 indicating that they had not attended the information and discussion session. Reasons for non-attendance are provided in Table 5. One mother wrote that her son had asked her not to attend:

*My son was a little embarrassed by the subject matter being shown and discussed and requested that I did not attend.*

**Table 5:** Parents/guardians reasons for not participating in the information session

Reason for not attending	Number (%)
I was unable to attend due to other commitments	15 (68 %)
It was not at a suitable time of day	4 (8 %)
I did not know about it	2 (9 %)
I did not need information on how to talk to my child about avoiding teenage pregnancy	1 (5 %)
I thought it might be embarrassing	1 (5 %)
It did not interest me	1 (5 %)

Mothers attending one of the focus groups directly following a session hypothesised about why other parents may not have attended:

*R: Why do you think other parents didn’t come?*

*M1: [They] can’t talk about it*.

*M2: Probably they didn’t have, part of it, didn’t have the time, part of it’s time, working…*

*M1: Can’t talk about it, don’t … can’t deal with it*.

*M2: And, again, as you said [indicating M1], for some, it’s just ‘bury your head in the sand’ type thing*.

*M1: Yeah, they don’t want to talk about it*.

*M3: Or they’ve already talked about it with their child and they don’t feel the need to go down that route again.* (Parent focus group, Catholic maintained secondary)

## Discussion

Overall recruitment rates in the current study were similar to those experienced in other UK-based sexual-health trials [20, 27]. While we met our school recruitment targets, we struggled to recruit grammar schools and encountered barriers due to prior commitment to other research and concerns about the possible burden on staff and pupils. Pupil recruitment targets were met with an overall pupil recruitment rate of 80.5 %. Absentees who did not return completed baseline questionnaires accounted for non-participation by 9 %, and parental and pupil opt-outs for the remaining 10 % (6.8 % and 3.6 %, respectively). We failed to reach the proposed target of having one parent/guardian representative for 50 % of participating pupils attend the parents’ information and discussion session. The following lessons learned relating to the barriers and facilitators of recruitment may be of benefit to those involved in similar trials.

### Facilitators to recruitment

**Fig. 2:**
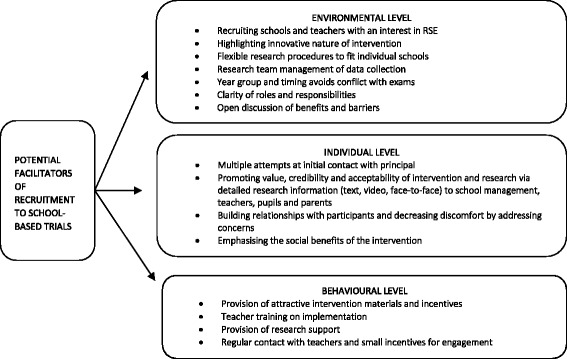
Potential facilitators to recruitment in school-based trials. *RSE* relationship and sexuality education

We have summarised facilitators to recruitment that emerged in the current study in Fig. 2. We took guidance from social learning theory [30] and its premise that people will choose to act in ways that they believe will offer them the maximum number of good outcomes and the minimum number of bad outcomes, and Lytle, et al. [11], who planned their school recruitment efforts to directly target a number of individual, environmental and behavioural factors posited to encourage participation. We suggest that these factors should be targeted in future RCTs.

#### Facilitating school recruitment

Targeting environmental-level facilitators involves ensuring that external obstacles to recruitment (such as schools with a lack of interest in RSE) are minimised. In particular, we found the following to be very important environmental facilitators of recruitment: approaching schools attending RSE training days, highlighting the innovative nature of the intervention, flexibility in terms of how and when the research was conducted in individual schools, the provision of support to schools by facilitation of the project by dedicated researchers, providing a clear outline of the roles and responsibilities of the school (and research team) from the outset, and facilitating discussion on the benefits and perceived barriers to taking part.

Individual-level facilitators, such as promoting the social benefits and credibility of the research aims, help school decision-makers recognise the importance of the research projects goals and objectives. We found that recruitment presentations by the research team using video testimonials from participants who took part in the pilot study and face-to-face contact with school management and teachers were important in this regard.

Finally, in targeting behavioural factors, we aimed to reduce the burden on schools and encourage school management and teachers to believe that participation in the research would be both manageable and rewarding for them. We did this by providing small incentives in the form of training, research and intervention materials, and support during implementation. For schools randomised to the control group, intervention materials were provided at the end of the trial. Additionally, providing refreshments during focus groups and meetings was appreciated, as were personal thank-you notes and small gifts to all involved. Although not used in this feasibility trial, we believe that monetary incentives of around £1000 would have had an impact on school recruitment.

#### Facilitating pupil recruitment

At an environmental level, researchers should highlight the innovative and engaging nature of the intervention to pupils. Equally, some pupils were as concerned about potential disruption to exams, as were school management and parents. A future trial might attempt to minimise the number of absentees by ensuring that data collection does not take place at a time when absences are more likely (e.g. Monday mornings, Friday afternoons and the weeks before Christmas or summer holidays). Similarly, when there are large numbers of absentees, researchers might offer to facilitate supervision and distribution of these at a particular time so as to encourage completion and ease the burden on the teacher.

At an individual level, researchers should ensure that data collection documentation is clear to parents and pupils, perhaps involving steering group members in ensuring clarity. Additionally, as suggested by Belzer, et al. [31], parents might be given the option of contacting the research team or school co-ordinator via phone or email for clarification prior to consenting. We believe that ensuring that pupils are provided with adequate information about their roles and responsibilities, and given an opportunity to meet with the research staff before data collection will also be beneficial to pupil recruitment. As none of the schools took up the offer of the trial manager to provide this on a separate occasion prior to data collection, this might be best achieved by creating a short video that teachers could show to pupils before the data collection session. Additionally, using a large hall when soliciting the consent of pupils to participate saves on resources in terms of the numbers of research assistants needed to attend the data collection sessions, we found that in most cases it was not an appropriate option for recruitment. When pupils decided they did not want to take part, this generally resulted in several pupils in the same area deciding not to take part.

Finally, in relation to facilitating pupil participation at a behavioural level, we suggest that researchers ensure they use engaging research and intervention materials. Of particular value in the current study was the use of new technologies including film and online surveys [23, 24]. The provision of small incentives in the form of one or two chocolates distributed at the end of questionnaire completion as a means of thanking pupils was also appreciated.

### Facilitating parent recruitment

As noted, we failed to reach the proposed target of parents and guardians recruited to the trial. As has been the experience of other researchers, the co-ordinators and principals in all the intervention schools indicated that parents were difficult to engage in general. Some of the often reported predictors of parental attendance include socio-demographic variables, such as education and income level, that may be related to resources and ability to attend [32–35]. Impacting such behavioural-level barriers might involve providing parents with incentives to attend, including funds for travel or child-minding.

Targeting individual-level factors, such as perceptions of the value of the intervention, might also be of benefit. For example, researchers have found that parents who think that an intervention may help address their children’s problems are more likely to engage [35]. Future trials might also consider developing a short video for parents that explains the potential significance of the trial and an intervention to help teenagers avoid pregnancy and the impact that an unintended pregnancy might have on their lives. While we had only modest success with the YouTube video, it was more successful than inviting parents to attend the session (12.5 % watched the video while only 2.3 % attended the parents’ information session). However, 12 % remains a low participation rate, given we initially estimated that 50 % of parents would attend the school session. Additionally, we were unable to collect information on how many parents actually watched the video in its entirety. Considering that the video was a rather lengthy 6 minutes, it is unlikely that most participants watched the whole video. With that in mind and building on the research of others (e.g. [36]), future trials might develop and examine the value of online and mobile technology further, for example by creating a more engaging video using animation or similar.

Our tentative conclusion based on the interviews with teachers and parents is that it is questionable whether or not parents/guardians actually want to be involved in RSE with their adolescent children or whether they would prefer to leave this in the hands of the school. It could be argued that high parental consent rates coupled with such low parental engagement might be a further indicator of this. Therefore, it may be more apt in such trials to reach out to parents with pertinent information, rather than seek their presence and involvement at the school, thereby facilitating engagement at both the individual and environmental levels.

## Conclusions

Poor recruitment to RCTs can result in underpowered and biased studies that fail to detect important effects and may require significant investment in extra resources. Successful recruitment is central to a trial’s success and dependent on careful planning and co-ordination by the whole research team. This article reflects on the methodological challenges of recruiting to a school-based sexual-health trial and the strategies we adopted to address them. We acknowledge the limitations of reflective articles such as this and the importance of future research that takes systematic approaches to examining and addressing recruitment to school-based trials. However, given the dearth of published information on optimal recruitment strategies for school-based research, we anticipate that our experiences and the lessons presented here will be helpful for those conducting similar trials.

## Abbreviations

CG, Catholic grammar school; CMS, Catholic maintained secondary school; NI, Northern Ireland; NIHR, National Institute for Health Research; RCT, randomised controlled trial; RSE, relationship and sexuality education; VOM, voluntary other-managed school
